# Monthly Memoranda

**Published:** 1853-11

**Authors:** 


### EDITORIAL DEPARTMENT.

Monthly Memoranda.—All physicians know well the difficulty of conducting successfully sevvre cases of dysentery. The various modes of treatment, lauded at different times and places, owe their success, undoubtedly, in a great measure, to the varying epidemics, and to the occasional modifying influences, under which the disease may present itself. Almost every physician has his own method of treatment. In the Ohio Med. Journal, Dr. S. C. Mendenhall thus sets forth his. "Ordered him to take a powder, composed of half a grain of morphia, two grains of ipecac, and three grains of tannin, every six hours. A thorough application of a saturated solution of camphor in sps. turpentine, over the abdomen, every two hours; an injection of creosote, suspended in water, three drops to the ounce, once in four hours, till the fœtor of discharges was corrected, then to be replaced by enemata of acet. lead in mntton broth. No cold water allowed—mutton broth the only drink." Dr. M. took this case, under very unpromising auspices; the patient had been the round of Botanic nonsense, till "composition" and "lobelia" had done their worst. But under the above treatment he improved, so that in eight days he sat up in his chair. The creosote was used in this case, from an uncommon apparent tendency to degeneration of thc tissues. A too frequent error, doubtless is to treat the disease, as dysentery, without sufficient study of all the accompanying modifying circumstances.

*Pustulation* is taught us in a new, but probably very useful method by the Nashville Journal. Hear him:

"Every physician knows the value of pustulation—every one knows that Tartar Emetic Ointment, Ipecac Ointment, and Croton Oil will produce it. The tartar emetic pustule often goes *deeper* that you desire—the croton oil pustulates a larger space than you "contracted for," and it is difficult to renew the crop with it. Like the tartar emetic pustule it is often painful and sometimes *fiery*, while the Ipecac is always uncertain. Now a *bacon rind* never fails. It produces *no* pain. The pustules may be wiped off, which is pleasant to the patient, and the rind be immediately applied. Next day there is a new crop for the reaper, which should be again wiped off and the rind reapplied. An old woman taught us this, bntit is not the less knowledge on that acconnt. Take a piece of old bacon rind as large as the part you desire to pustulate—trim the fat pretty well off the inside—have it nicely washed with soap and water, and confine it to the part. The same piece will answer for an indefinite period. Try it."

*Butter*, we have always thought, is not the poisonous and villanous compound some so strenuously insist upon. The only objection we have to it, is its price. But this does not concern the properties of which we intended to speak. It is composed as every one knows, of the fat-globules of the milk, which is in fact a transparent liquid, holding in suspension these numerous globules which rise to the surface when fresh milk is at rest. In churning, the membranes of the oil globules are ruptured, which latter then unite together and butter is formed. It seems most reasonable, therefore, that good butter should be eminently nutritious: that it might, used in proper quantities, act upon the system as does cod liver oil, by restoring and building up the tissues, and thus be a very useful restorative, in degeneration of the structures, and tubercular formations. The only objection attending its abundant use is, that it is sometimes difficult of digestion. But why not have a medicated butter, and make sickness as much a luxury as possible? M. Trousseau suggests the following, as a very useful combination. Fresh butter, four ounces: iodide potassium three fourths grain; bromide of potaassium, three grains, common salt, half ounce; to be used on *thin* slices of bread. We cannot testify personally to this mixture, but we would earnestly suggest the propriety of a liberal use of this nutritious substance, and a combination of appropriate medicinal agents with it.

*Veratrine in Rheumatism.*—Some marvelous results have followed the use of Veratrine in articular diseases. The trial was made by M. Trousseau, who gave the tenth of a grain in a pill the first day; two on the seeond; three on the third, and so on increasing the dose, one pill each day, until it reached six or seven. Its actions appears to be most decided in breaking down the inflammatory action. It is maintained that it has anti-rhuematic virtues and from its influence over the pulse it may be found, by further trial, to be a most valuable therapeutic agent in all these arthrodial affections.

*Suphuric ether in Colic.*—The editor of the Philadelphia Med. and Surgical Journal, Prof. Bryan, cured permanently a dangerous case of colic in a lady by the inhalation of sulphuric ether. Good cloroform would doubtless answer equally well in cases of spasmodic colic.

*Enteric Fevers.*—When the tongue is loaded and brown, and tympanitic condition of the abdomen, the emulsion of turpentine is a most valuable therapeutic agent. In this condition there is usually delirium, the secretion by the kidneys suspended, or very much diminished, and the skin either in a negative state, or dry and husky, the pulse small and frequent. Under such circumstances, the oil of turpentine in doses of fifteen drops in simple syrup, given every two or four hours, will prove highly valuable. To aid still further the depurators in throwing off the morbific poison freely diffused through the system, the solution of the chlorate of potass made by dissolving fifteen grains in half an ounce or any convenient amount of water. This amount to be taken every four or six hours, followed by a glass of water, acidulated with a few drops of hydrochloric acid to neutralize the salt. The effects will be that the skin will become warm, the tongue will lose its dark shade, and the amount of urine will be largely increased. The capillary circulation will be increased and the depuratory powers of the kidneys largely promoted. By some it is thought that oxygen is also supplied to the blood by it. From our experience in the treatment of this form of fever we feel that we cannot too strongly urge the consideration of these subjects upon the attention of the profession. Quite a number of these cases were treated in this locality this season, in which, after the use of mercurials at first, these remedies, with the occasional use of pulv. ipecac. comp., constituted those upon which the chief reliance was placed, and in every case almost where the treatment was persisted in, a recovery followed.

*Nocturnal Perspirations.*—In the exhausting sweats attending phthisis, the tannate of quinia, in six or eight grain doses, has been found highly valuable. These exhausting sweats often follow, in this country, a long course of intermittent in which cases the *tannate* would be indicated. It would be advantageously given in cold infusion of garden sage (salvia.)

*Measles.*—Dr. Walz has cured 343 cases of rubeola with the frictions with fat. There were 57 cases very unpromising, but all recovered. He has also treated a number of cases of scarlatina with like success.

*Overdose of Chloroform.*—As soon as indications of excessive anæsthesia is perceived, the tongue should be drawn out so as to allow the ingress of air. This can be done either with the finger or the curved spatula. Then dash the face with cold water. If this fail to resuscitate, interrupted pressure upon the chest so as to exclude any remaining vapour. This can be aided by introducing a tube into the larynx and inflate the lungs by a very gentle strain of air. This is to be followed by compression and anon renewing the inflation, and continue this while there is a hope of a revival to consciousness.

